# Pooling sputum samples for the Xpert MTB/RIF assay: a practical screening strategy for highly infectious tuberculosis cases

**DOI:** 10.1186/s12879-024-09020-w

**Published:** 2024-01-23

**Authors:** Jianfeng Zeng, Huan Huang, Xuhui Liu, Zhen Huang, Weijian Liu, Houming Liu, Shuihua Lu

**Affiliations:** 1 Shenzhen Third People’s Hospital, National Clinical Research Center for Infectious Disease, 518112 Shenzhen, China; 2 Shanghai Key Laboratory of Atmospheric Particle Pollution Prevention (LAP3), Department of Environmental Science & Engineering, Fudan University, 200438 Shanghai, China

**Keywords:** Tuberculosis, Xpert, Pooling assay, Active screening, Infectivity

## Abstract

The Xpert MTB/RIF test (Xpert) can help in the accurate screening of tuberculosis, however, its widespread use is limited by its high cost and lack of accessibility. Pooling of sputum samples for testing is a strategy to cut expenses and enhance population coverage but may result in a decrease in detection sensitivity due to the dilution of *Mycobacterium tuberculosis* (*Mtb)* by sample mixing. We investigated how the mixing ratio affected the detection performance of Xpert. We used frozen sputum samples that had been kept after individual Xpert assays of the sputa from *Mtb*-confirmed TB patients and non-TB patients. Our results showed that the overall sensitivity of the Xpert pooling assay remained higher than 80% when the mixing ratio was between 1/2 and 1/8. When the mixing ratio was raised to 1/16, the positive detection rate fell to 69.0%. For patients with either a high sputum *Mtb* smear score ≥ 2^+^, a time-to-positive culture ≤ 10 days, or an Xpert test indicating a high or medium abundance of bacteria, the pooling assay positivity rates were 93.3%, 96.8%, and 100% respectively, even at a 1/16 mixing ratio. For participants with cavities and cough, the pooling assay positivity rates were 86.2% and 90.0% at a 1/8 ratio, higher than for those without these signs. Our results show that the Xpert pooled assay has a high overall sensitivity, especially for highly infectious patients. This pooling strategy with lower reagent and labor costs could support TB screening in communities with limited resources, thereby facilitating reductions in the community transmission and incidence of TB worldwide.

**Supplementary Information:**

The online version contains supplementary material available at 10.1186/s12879-024-09020-w.

## Introduction

Tuberculosis (TB), a global epidemic caused by *Mycobacterium tuberculosis* (*Mtb*), is a leading cause of illness and death worldwide [1]. Although the World Health Organization (WHO) launched its ambitious “END TB” plan about a decade ago, progress has been sluggish in reducing TB-related mortality and morbidity. This is partly due to the fact that up to 30–40% of TB patients are undiagnosed [1, 2]. Individuals without typical clinical signs and symptoms or those without access to healthcare, especially in resource-limited communities, are more likely to experience underdiagnosis and might spread the disease among households and communities, preventing decreases in TB-related illness and deaths.

Actively screening and treating these concealed cases is an ideal strategy to eliminate TB. And yet, due to the lack of suitable screening techniques, screening for TB in large populations is highly challenging. Symptom screens and chest X-ray radiography (CXR) are the most used screening methods [3], but they have limited specificity in predicting TB, and do not directly reflect infectivity. Xpert MTB/RIF test (Xpert), a WHO-recommended rapid diagnostic test for TB, typically exhibits higher accuracy (sensitivity plus specificity) than symptom screens and chest X-rays [4]. To date, however, the value of Xpert for TB screening in large populations remains limited, primarily due to (1) the high cost of Xpert cartridges for a single test, (2) the low cost-effectiveness due to the fact that the majority of tests return negative results, and (3) the time- and labor-intensity due to insufficient lab technicians and instruments. These shortcomings are particularly evident in areas where resources are limited, but where extensive or continuous monitoring is required.

Testing of pooled sputum samples is a feasible cost-saving strategy to facilitate TB screening of large populations [5]. Usually, sputa from several individuals are mixed into a pool for testing, and a negative test result can rule out TB infection. If the result is positive, all samples are retested separately to determine which patients are TB-positive. By combining samples, this assay’s benefits include increased testing efficiency but, more importantly, reduced testing costs. The most noticeable disadvantage is the possibility of a decrease in detection sensitivity due to the dilution of *Mtb* by sample mixing. A mixing ratio that is too high may significantly impair Xpert’s sensitivity due to TB’s typical paucibacillary nature, thus limiting its value for TB screening in a large population.

To investigate the effect of pool size on the sensitivity of the Xpert assay, we mixed sputum samples from bacteriologically confirmed TB patients with sputum samples from non-TB patients in different ratios (from 1/2 to 1/16) for the Xpert assay. The effects of the dilution ratio on the overall rate of detection and the influence of estimated *Mtb* loads were assessed.

## Methods

### Sample collection

All participants included in this study were outpatients with presumptive TB who underwent Xpert testing between July and October 2022. We collected 3–5 mL of spot sputum from each recruited patient, added reagents equal to the volume of sputum, and tested with the Gene Xpert MTB/RIF kit according to the manufacturer’s instructions. Whether the Xpert test was positive or negative, the samples with a remaining volume of more than 2 mL were frozen at -80 °C and included in the study. Patients were allocated into an Xpert-positive group (TB group) and an Xpert-negative group (non-TB group) based on the Xpert results. Patients in the non-TB group were only included after a rigorous clinical evaluation that included CXR, sputum culture and smear, and responsiveness to antituberculosis chemotherapy. The samples from the non-TB group were thawed and mixed to a pool (non-TB samples pool, NSP). The demographics and diagnostic information of TB patients were collected. Shenzhen Third People’s Hospital’s Ethics Committee approved this study (No. 2022-008).

### Pooling assay

**Fig. 1:**
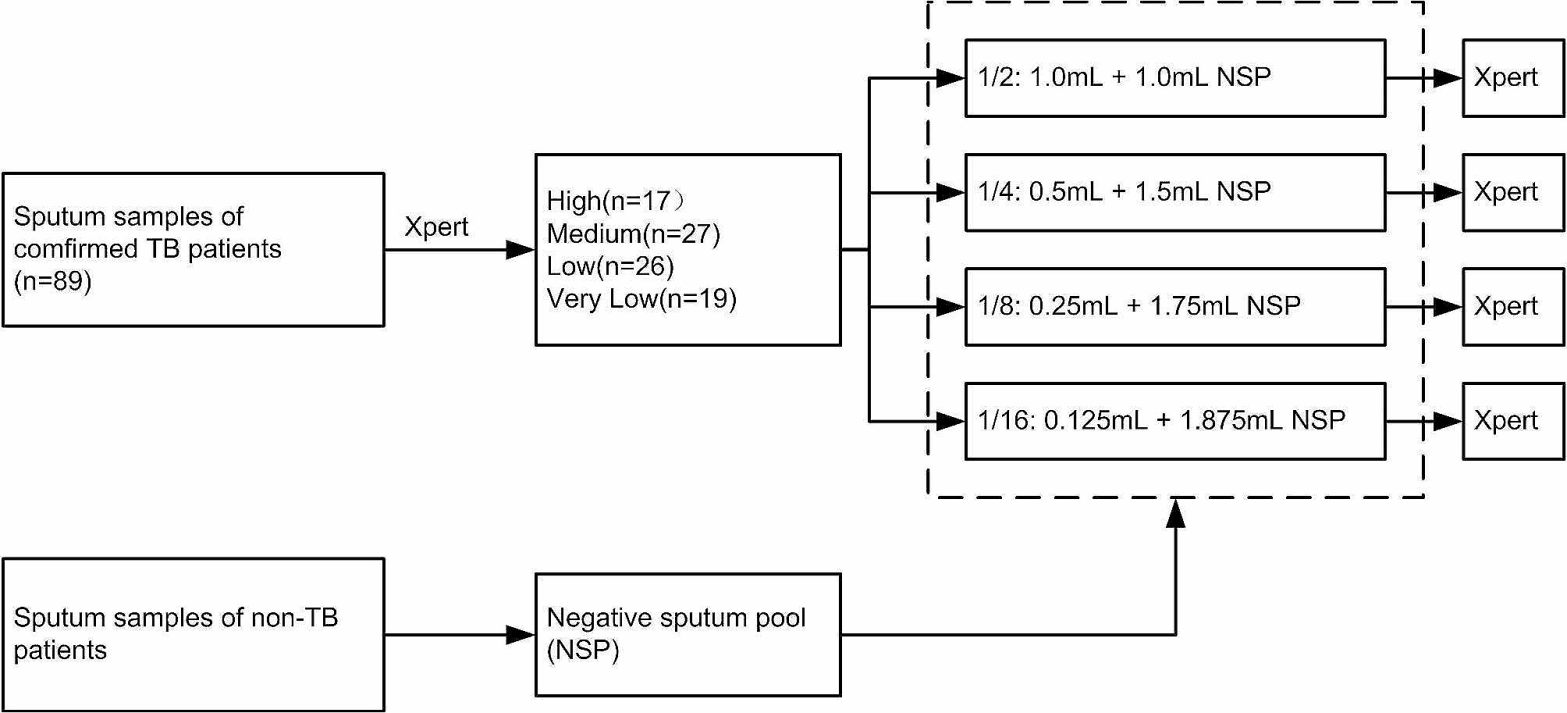
Flow chart of the Xpert pooling assay of frozen samples from confirmed TB and non-TB patients

Samples were thawed on the day of the assay. The NSP was used to serially dilute the Xpert-positive samples. A 1/2 ratio mixed sample was obtained by adding 1 mL NSP to 1 mL Xpert-positive sputum, then two-fold dilutions of this to 1/4, 1/8, and 1/16 were made in NSP. The dilutions were stirred with a vortex mixer and then added to the Xpert assay cartridge (Fig. 1).

### Statistical analysis

Following the manufacturer’s guidance, the *Mtb* load of the samples was defined as high, medium, low, and very low when the CT values of Xpert were < 18, 18–22, 22–28, and > 28, respectively. The positivity rate of the Xpert pooling assay was calculated variously after including all patients, patients with or without cavities and cough, with diverse Xpert and smear grades, and time-to-positive (TTP) of cultures. These data were visualized by GraphPad 8.0.2.

## Results

Residual sputum samples were collected from 87 TB patients who had positive Xpert tests (Table 1) and 281 non-TB patients (Table S1). Of these, 74.7% were male, 55.2% were middle-aged people (between the ages of 30 and 60), and 98.9% were without HIV infection. The CXR of these individuals indicated that 74.7% had cavities in their lungs. In addition, 80.5% of participants had a significant cough. The proportions with high, medium, low, and very low *Mtb* loads defined by the CT values of Xpert were 19.5%, 31.0%, 30.0%, and 19.5%, respectively. All samples in the group with a high *Mtb* load had a smear grade of 2^+^ or higher, as compared to 26.9% and 23.6% in the low and very low groups, respectively. In the high and medium groups, mycobacterial cultures were 100% positive, while in the low and very low groups, they were 77.0% and 76.5% positive, respectively.

**Table 1:** Demographics and diagnostic information of TB Patients

Characters			Xpert			
		Total(%)	High(%)	Medium(%)	Low(%)	Very Low(%)
		*n* = 87	*n* = 17	*n* = 27	*n* = 26	*n* = 17
**Male**		74.7	94.1	77.7	61.5	70.5
**Age**	< 30	18.4	17.6	14.8	19.2	23.5
	30–60	55.2	52.9	63.0	50.0	52.9
	> 60	26.4	29.4	22.2	30.8	23.6
**PLHIV**		1.1	0.0	0.0	3.8	0.0
**Xpert**	High	19.5	100.0	NA	NA	NA
	Medium	31.0	NA	100.0	NA	NA
	Low	30.0	NA	NA	100.0	NA
	Very Low	19.5	NA	NA	NA	100.0
**Cavity**	With	74.7	88.2	88.9	50.0	76.5
	Without	20.7	11.8	7.4	42.3	17.6
	NA	4.6	NA	3.7	7.7	5.9
**Cough**	With	80.5	100.0	81.5	50.0	64.7
	Without	18.4	0.0	14.8	42.3	35.3
	NA	1.1	NA	3.7	7.7	NA
**Smear**	Negative	29.9	0.0	11.1	57.7	47.1
	Scanty	2.3	0.0	0.0	3.8	5.9
	1+	11.5	0.0	11.1	11.5	23.5
	2+	11.5	5.9	14.8	19.2	NA
	3+	10.3	17.6	18.5	0.0	5.9
	4+	29.9	64.7	40.7	7.7	11.8
	NA	4.6	11.8	3.7	0.0	5.9
**Culture**	Negative	8.0	0.0	0.0	11.5	23.5
	Positive	85.1	88.2	96.3	77.0	76.5
	NA	6.9	11.8	3.7	11.5	NA

n: number of participants with positive results; PLHIV: Patients with HIV infections

**Fig. 2:**
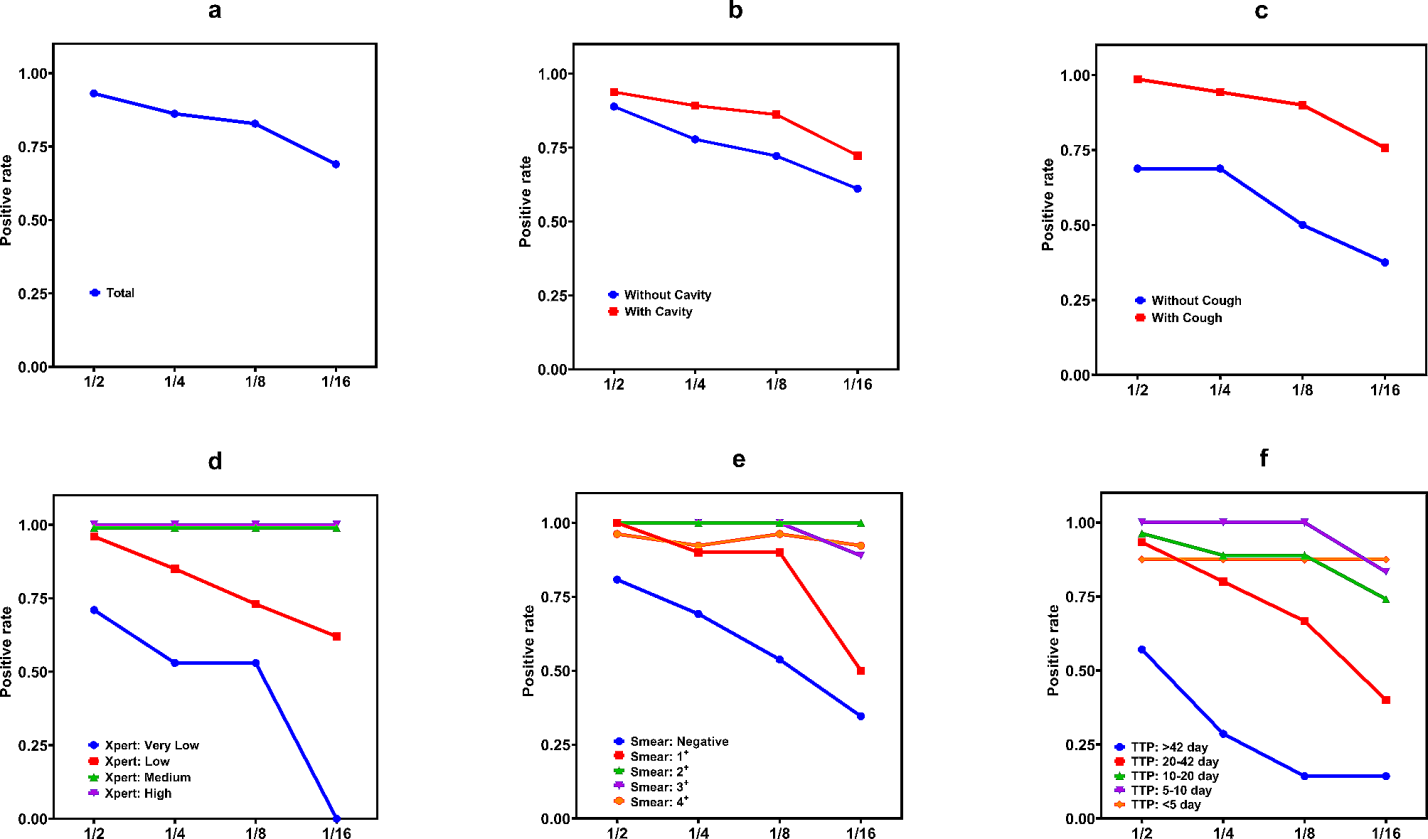
Positivity rates of Xpert pooling assays for the diagnosis of tuberculosis. (**a**) All patients. (**b**) Patients with (*n* = 18) or without cavitation (*n* = 65 ). (**c**) Patients with (*n* = 70) or without cough (*n* = 16). (**d**) Patients with very low (*n* = 17), low (*n* = 26), medium (*n* = 17), and high (27) *Mtb* load defined by the CT values from Xpert. (**e**) Patients with negative (*n* = 26), 1^+^ (*n* = 10), 2^+^ (*n* = 10), 3^+^ (*n* = 9), and 4^+^ (*n* = 26) grade of smear. (**f**) Patients with < 5 (*n* = 8), 5–10 (*n* = 24), 10–20 (*n* = 27), 20–42 (*n* = 15), > 42 (*n* = 7) time-to-positive culture (TTP).

The overall positive rate of the pooling test was 82.8% when the mixing ratio was less than 1/8. When the mixing ratio was raised to 1/16, the positive detection rate fell to 69.0% (Fig. 2a). Positive rates of the pooling tests were higher in patients with cavities or cough than in those without, with positive rates of 86.2% (with cavities) and 90.0% (with cough) at a mixed 1/8 ratio, respectively (Fig. 2b and c). For patients with medium or high bacterial load indicated by Xpert, the pooling assays yielded 100% positivity even at a 1/16 mixing ratio (Fig. 2d). In sputum samples with a smear score of 2^+^ or higher, the pooling assays were 97.7% positive at 1/8 mixture and 93.3% positive at 1/16 mixture (Fig. 2e). When the TTP of culture was less than 10 days, the positive rate of the mixed test was greater than 96.8% at 1/16 mixture (Fig. 2f).

## Discussion

Our research shows that the overall sensitivity of the Xpert assay remained higher than 80% when the mixing ratio was between 1/2 and 1/8, which is comparable to the positive rates of several earlier studies of sputum sample pooling (89.1–91.8%) [5–7]. More notably, we observed that patients with high sputum *Mtb* load (smear ≥ 2^+^, TTP ≤ 10 days, and Xpert medium or high), cough, or cavities had a higher positive rate by the Xpert pooling assay, even at a 1/16 mix ratio. This implies that at certain mixing ratios, Xpert maintains a high level of sensitivity while effectively screens out almost all patients who are considered to be at high risk of transmission. When resources are limited, it may be more cost-effective to prioritize pooled screening to find and treat highly infectious cases in order to reduce the community transmission of tuberculosis; the public health value of Xpert pooling assay in a large population could be very substantial. The lower reagent and labor costs being incurred, particularly when positive results are infrequent may permit such screening to be repeated regularly for timely detection of TB in the community.

The Xpert assay has a low limit of detection (LOD) at 131 cfu/mL. This is significantly better than 10,000 cfu/mL for smears and very close to the LOD for solid culture [4]. Xpert, therefore, maintains a comparatively high sensitivity even after the sample has been diluted. However, the use of the Xpert pooling assay to detect patients who have low *Mtb* load (Xpert low or very low and smear less than 1^+^) remains a problem due to inadequate sensitivity in this population, as demonstrated here. None of the samples with a very low *Mtb* load tested positive when sputum samples were mixed at a ratio of 1/16. Even with a 1/4 mixing ratio, only 53.0% of samples were positive. This inadequate sensitivity at low loads is consistent with Lao’s finding that pooled tests identified 40% of TB patients (2/5) with very low *Mtb* loads at a 1/4 mixing ratio [6]. The next-generation version of Xpert, Xpert MTB/RIF Ultra (Xpert Ultra), has improved sensitivity (LOD 15.6 cfu/mL [8], close to that of liquid culture); in the pooling strategy it could give results 100% consistent with individual detection. The use of the Xpert Ultra may offset the reduction of sensitivity caused by pooling samples with very low *Mtb* loads, but the increased cost per sample may make the Xpert Ultra pooling test less attractive to countries with limited resources. The balance between sensitivity and cost of testing by pooling assay should be carefully assessed alongside the prevalent TB characteristics before the screening of large populations begins.

There are several limitations to this study. First, this is merely a lab simulation of a population-based pooling test in which negative and positive sputum samples are simply mixed in various ratios. Our study’s findings, however, may help to guide the selection of mixing ratios based on estimates of the proportion of infectious cases in the community. Second, we only focused on how different mixing ratios affected the precision of the mixed test in our study. The time and money to be saved by mixed testing also depend on the level of TB prevailing at the screening location, with smaller mixing ratios at higher prevalence rates being more cost- and time-effective than larger mixing ratios [9].

In conclusion, our results show that the Xpert pooled assay has high overall sensitivity, especially for highly infectious patients. This pooling strategy with lower cost and labor consumption could support TB screening in communities with limited resources, thereby reducing the community transmission and incidence of TB worldwide.

### Electronic supplementary material

Below is the link to the electronic supplementary material.

Supplementary Material 1

## Data Availability

The datasets used and/or analysed during the current study available from the corresponding author on reasonable request.

## Abbreviations

*Mtb*: *Mycobacterium tuberculosis*
TB: Tuberculosis
WHO: The World Health Organization
CXR: Chest X-ray radiography
Xpert: Xpert MTB/RIF test
NSP: Non-TB samples pool
TTP: Time-to-positive
LOD: Low limit of detection
Xpert Ultra: Xpert MTB/RIF Ultra
