# Supplementary material for: Pooling sputum samples for the Xpert MTB/RIF assay: a practical screening strategy for highly infectious tuberculosis cases
Source: BMC Infect Dis. 2024 Jan 23;24:122. doi: 10.1186/s12879-024-09020-w (PMC10807086; doi:10.1186/s12879-024-09020-w)
Supplement: Supplementary file 1 — Supplementary Material 1 [file 12879_2024_9020_MOESM1_ESM.doc]

**Supplementary Materials for**

Pooling Sputum Samples for the Xpert MTB/RIF Assay: a Practical Screening Strategy for Highly Infectious Tuberculosis Cases

Table S1. Demographics and diagnostic information of non-TB Patients

| Characters |  |  | Total(%) |
| --- | --- | --- | --- |
|  |  | n |  |
|  |  |  |  |
| Sex | Male | 165 | 58.7 |
| Age | <30 | 21 | 7.5 |
|  | 30-60 | 141 | 50.2 |
|  | >60 | 119 | 42.3 |
| PLHIV |  | 0 | 0 |
| Xpert | Negative | 281 | 100.0 |
| Cavity | With | 32 | 11.4 |
|  | Without | 249 | 88.6 |
| Cough | With | 229 | 81.5 |
|  | Without | 52 | 18.5 |
| Smear | Negative | 281 | 100.0 |
| Culture | Negative | 281 | 100.0 |

n: number of participants with positive results; PLHIV: Patients with HIV infections.
